# Optimising Cold Spray Additive Manufacturing: Pressure-driven enhancement of mechanical performance in copper deposits

**DOI:** 10.1371/journal.pone.0353072

**Published:** 2026-07-02

**Authors:** Steven Camilleri, Thi Thuy Tien Tran, Keita Nomoto, Andrew Duguid, Matthew Harbidge, Riyan Rashid, Kannoorpatti Krishnan, Naveen Kumar Elumalai

**Affiliations:** 1 Advanced Manufacturing Alliance, Energy and Resources Institute, Charles Darwin University, Darwin, Australia; 2 SPEE3D, Charles Darwin University, Casuarina NT, Australia; 3 School of Aerospace, Mechanical and Mechatronic Engineering, Faculty of Engineering, The University of Sydney, Sydney, NSW, Australia; King Mongkut’s University of Technology North Bangkok, THAILAND

## Abstract

Cold spray additive manufacturing (CSAM) is a solid-state process capable of producing dense metallic components without melting, making it highly attractive for copper applications requiring both electrical conductivity and mechanical integrity. In this study, the influence of spray pressure at 30 bar, 40 bar, 50 bar and 60 bar on particle velocity, microstructure, and properties of cold-sprayed copper was systematically investigated using a LightSPEE3D system. The cold spray deposits were characterised by X-ray diffraction (XRD), hardness testing, eddy current conductivity, tensile evaluation, and scanning electron microscopy (SEM). The results reveal that increasing spray pressure enhances particle velocities beyond the critical threshold for copper, leading to improved inter-particle bonding and microstructural refinement consistent with severe plastic deformation and possible continuous dynamic recrystallisation (cDRX)-assisted mechanisms. XRD analysis suggested progressive crystallite refinement and increased dislocation density with pressure, which directly correlated with improved ductility. While hardness decreased due to recovery and recrystallisation, electrical conductivity increased, likely due to improved inter-particle continuity and reduced interfacial discontinuities. Tensile testing showed a clear strength–ductility transition, with deposits at higher pressures exhibiting substantially improved ductility approaching bulk copper behaviour and fully ductile fracture morphologies. Overall, the findings identify an optimum processing window at higher spray pressures, where copper cold spray deposits achieve a balanced combination of conductivity, ductility, and strength. This study highlights the critical role of spray pressure in controlling the interplay between particle velocity, dynamic recrystallisation, and multifunctional performance in CSAM copper components. This study establishes a process–structure–property relationship linking particle velocity, XRD-derived microstructural evolution, conductivity, and tensile behaviour within a LightSPEE3D CSAM system.

## 1. Introduction

Cold spray (CS) technology is an advanced solid-state deposition process increasingly used in additive manufacturing (AM), including 3D printing, to fabricate metallic parts and coatings without melting the feedstock material. Unlike conventional thermal spray or fusion-based AM methods, cold spray propels powder particles to supersonic velocities using compressed gas, enabling them to plastically deform and bond upon impact with the substrate at relatively low temperatures. This low-heat process minimises oxidation, thermal distortion, and residual stresses, making it ideal for applications where microstructural integrity and high performance are critical [1–4].

In recent years, cold spray additive manufacturing (AM) has emerged as a promising technique for producing freestanding parts and for repairing or enhancing existing components in a layer-wise manner [4,5]. Cold spray AM offers high deposition rates, compatibility with a broad range of metals, alloys, and composites, and the ability to fabricate large or complex geometries through robotic nozzle control. This capability supports supply-chain resilience by enabling rapid local production or repair of components, which is especially valuable in industries facing long lead times or reduced casting/machining availability.

Recent studies in advanced additive manufacturing and metallic processing have demonstrated that processing conditions strongly influence microstructural evolution and resulting mechanical and functional properties [6,7]. A distinct advantage of cold spray lies in its non-melting nature during deposition. In this process, metal powder particles are accelerated through a convergent–divergent (De Laval) nozzle to velocities around two to three times the speed of sound before impacting the substrate, where they undergo intense plastic deformation and bond [1–4,8]. Commercial cold spray systems are generally divided into low-pressure cold spray (LPCS) and high-pressure cold spray (HPCS). LPCS systems operate below 0.6 MPa with particle velocities of 350–700 m/s, typically depositing softer metals and offering advantages in portability, simplicity, and cost [9–11]. HPCS systems operate at 2–4 MPa, producing particle velocities of 800–1200 m/s. These higher velocities broaden the range of depositable materials (including hard metals and alloys) and improve material density and adhesion, though at the cost of greater equipment complexity and energy requirements.

Spray pressure is one of the most critical parameters in the cold spray process, as it directly influences the velocity of powder particles accelerated through the spray nozzle. Higher spray pressures result in increased gas flow rates and therefore higher particle velocities upon impact with the substrate or previously deposited layers. This kinetic energy transfer governs the degree of plastic deformation, particle flattening, and bonding quality in the material [8,12,13]. The particle velocity must exceed a material-specific critical velocity for successful bonding to occur. Below this velocity, particles may rebound or adhere poorly, leading to materials with high porosity and weak interparticle cohesion. When the spray pressure is sufficiently high, particles impact substrates at velocities that induce severe plastic deformation, fracturing and breaking up surface oxides and contaminants, thus exposing fresh metal surfaces for metallurgical bonding. This process also triggers adiabatic shear and localised shear bands which further enhance bonding through interlocking and molecular mixing at the interface [13–15].

Furthermore, gas temperature and nozzle geometry in combination with pressure determine the flow dynamics that affect particle acceleration and thermal conditions, indirectly influencing deformation mechanisms and bonding strength [1,4]. High-pressure cold spray (HPCS) systems can accelerate particles up to supersonic velocities, while low-pressure systems are limited to lower velocities, often resulting in less dense and weaker [9,10].

The mechanical properties of cold-sprayed materials are strongly linked to their microstructural features that develop during high-strain-rate particle impact. The plastic deformation experienced by particles causes severe lattice distortions and grain refinement, producing ultrafine or nanocrystalline grains in the deposited material. Smaller crystallite size generally enhances mechanical strength due to grain boundary strengthening mechanisms (Hall-Petch effect) and improved hardness [4,16,17]. Thus, spray pressure plays a defining role by modulating particle velocity, which in turn governs the impact strain rate and plastic deformation necessary for effective bonding and mechanical performance.

While previous studies have investigated the influence of spray pressure on copper cold spray deposition, most reports focus primarily on deposition efficiency, coating adhesion, or isolated mechanical properties. A comprehensive understanding of how spray pressure influences the coupled evolution of particle velocity, microstructure, electrical conductivity, and tensile behaviour remains limited, particularly in high-deposition-rate CS systems. Furthermore, the transition regime between insufficient bonding and fully ductile behaviour has not been clearly identified for cold-sprayed copper.

The present study addresses this gap by establishing an integrated process, structure and property relationship linking particle velocity, XRD-derived microstructural evolution, conductivity, tensile response, and fracture morphology over a controlled pressure range (30–60 bar). Particular emphasis is placed on identifying the critical transition regime associated with bonding evolution and multifunctional property optimisation in CS copper deposits. The findings provide both mechanistic insight and practical processing guidance for industrial cold spray additive manufacturing applications. Unlike conventional optimisation studies focused on a single property, the present work evaluates the simultaneous evolution of conductivity, strength, and ductility to identify a multifunctional processing window for cold-sprayed copper.

### 1.1. Powder

**Fig 1:**
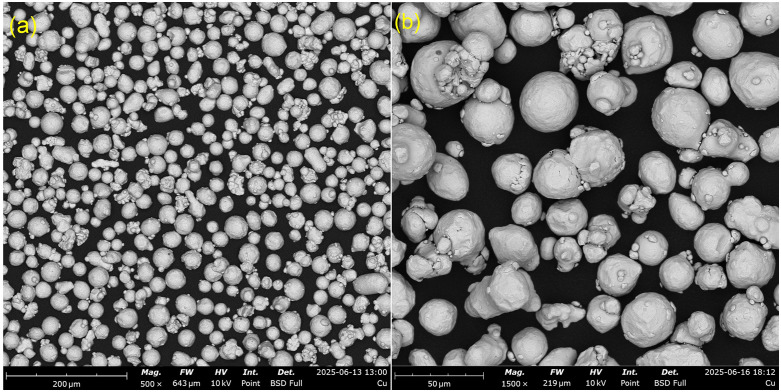
SEM image of Cu powder at different magnifications.

Copper powder was supplied by SPEE3D with a composition of Cu > 99%. Powder characterisation was provided by the Supplier as in Table 1. SEM images of Cu powder (Fig 1) show spherical morphology with satellite particles.

**Table 1:** Copper powder specification.

Material geometry	Hall flow (s/50g)	Angle of repose (dg)	Bulk density (g/ml)	Hausner Ratio	Composition	Size distribution
Spherical	14.1	42.09	4.86	1.11	99.95% Cu	D10: 17.56 µm D50: 26.77 µm D90: 39.50 µm

### 1.2. Cold spray process

Standard operating procedures and settings were used as supplied by SPEE3D. Deposition of the copper powder onto an aluminium substrate (Al-alloy 5000 series) was carried out using a LightSPEE3D CSAM printer [33]. Nitrogen gas was used as the working gas and employs a stationary cold spray nozzle, whilst a robotic arm moves the workpiece in accordance with a deposition strategy determined by a proprietary 3D slicing algorithm.

**Fig 2:**
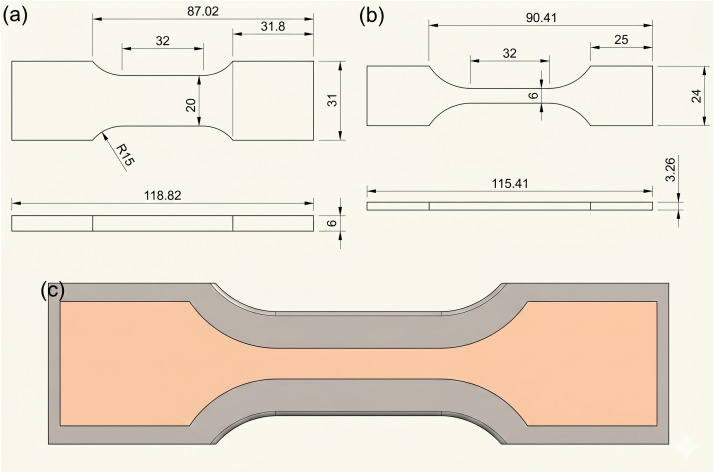
Tensile specimen geometry (a) As-sprayed drawing; (b) Machined tensile sample; (c) Image showing machined sample geometry within printed geometry.

To produce test tensile specimens, the powder was deposited onto aluminium substrates in horizontal direction with the design as shown in Fig 2. The samples were deposited using nitrogen as carrier gas was pressurised to 30, 40, 50 and 60 bar and preheated to 750 °C prior to entering the converging section. The selected pressure range (30–60 bar) was intentionally chosen to span conditions near, below, and above the expected critical velocity regime for copper deposition. This enabled identification of the transition from weak bonding behaviour to improved metallurgical bonding. The stand – distance was kept at 20 mm and the feed rate was 60g/s. The test specimens were machined and removed from the substrate for mechanical testing.

### 1.3. Velocity of powder particles out of nozzle measurement

An Oseir HiWatch CS2 was used to capture particle velocities exiting the barrel of the converging diverging nozzle. The device uses a pulsed laser to illuminate particles in the gas stream, and a camera captures an image. The laser pulses 3 times at a user-controlled interval that illuminates particles within the gas stream, and the exposure time of the camera is sufficient so that 3 instances of the particle can be seen in an image. The measurement area of the camera is 8 mm wide and 6 mm tall. The distance the particle has travelled, and the pulse interval of the laser are used to determine the velocity of the particles in the image. The particle velocities reported for each condition are the mean particle velocity measured during testing of each condition.

The HiWatch CS2 was installed within the build chamber of the LightSPEE3D CSAM printer. The following parameters were used for collecting the velocimetry data: Laser pulse interval of 0.4µs; Camera captures images at 30FPS; 200 images per data set; Powder feed rate of 2.2 cc/min. Measurement area center point positioned to 20 mm above barrel exit.

The Oseir soft ware was used to interpret the 200 images captured for each condition. The software uses the 3 illuminated points for each particle to determine the axial and longitudinal velocity components of the particle, its position within the spray beam, and estimates its size. A report can be generated by the software providing a summary of the particle data for each condition.

The reported values represent mean particle velocities. However, gas flow dynamics produce a spread in particle velocities that may influence bonding behaviour and deposition efficiency.

### 1.4. XRD testing of specimens

XRD was also conducted to observe the change in peak of each specimen using copper K_α_ radiation in Malvern Panalytical machine. The dislocation density was estimated using Williamson–Hall (WH) analysis.

The Williamson–Hall method separates size and strain effects:


$$\begin{array}{c} \beta .cos\theta =\frac{\kappa \lambda }{D}+\text{4}\varepsilon .sin\theta \end{array}$$

Where:

- β = Full width at half maximum (FWHM) (radians)
- θ = Bragg angle (radians)
- k = shape factor (0.9)
- λ = X-ray wavelength (Cu K_α_ = 0.154nm)
- D = crystallite size (m)
- ε = microstrain (%)

Dislocation density (ρ) relates to microstrain:


$$\begin{array}{c} \rho =\frac{\text{2}\sqrt{\text{3}\varepsilon }}{bD} \end{array}$$

Where:

- ε = microstrain from WH plot
- D = crystallite size (m)
- b = Burgers vector (for copper, b = 2.556×10 ^−10^ m)

The calculation was done on the main plane of Cu materials – (111) plane as it is the strongest and most intense diffraction peak for face-centered cubic (FCC) copper and easier to detect and analyse more accurately. It is also less prone to overlap with peaks from other phases or impurities compared to some higher-index planes.

### 1.5. Mechanical test and fracture morphology study

The hardness of the specimens was evaluated using an industrial Rockwell hardness tester using an HRB-scale. In addition, eddy current testing was performed to monitor variations in the electromagnetic field induced by eddy currents, enabling the detection of flaws and the assessment of material properties in the specimens (Info on the model and make of the eddy current testing instrument needed). Eddy current test device used was a Fischer Sigmascope SMP350.

- Samples and device are left in a climate-controlled room for several hours to reach temperature equilibrium.
- Calibration is checked using Fischer supplier calibration samples.
- Samples are tested in 4 locations, 3 times and the result is average.
- Test frequency used is 60kHz.

Although the testing procedure minimises variability, conductivity measurements may still be influenced by surface condition, local anisotropy, and probe positioning.

Tensile testing was conducted by an independent third-party laboratory. The fracture surfaces of the tensile specimens were examined using scanning electron microscopy (SEM) to analyse fracture morphology.

## 2. Results and discussion

### 2.1. Particle velocity

**Fig 3:**
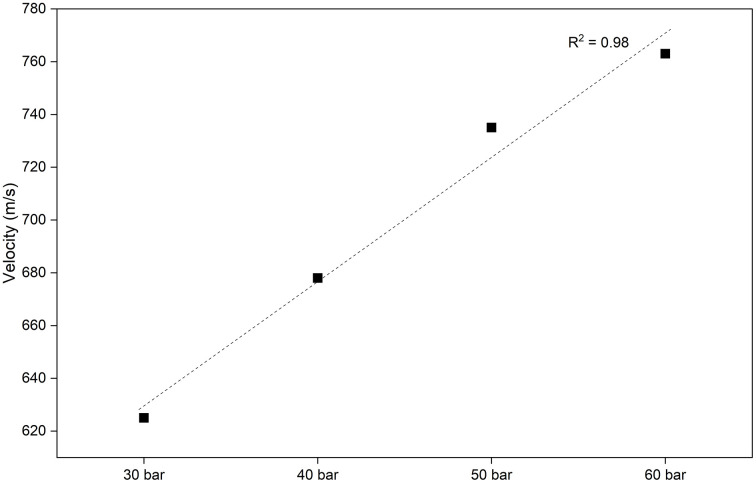
Correlation between particle velocity (m/s) vs nozzle pressure (bar).

Nozzle pressure critically influences particle velocity, with measurements in this study indicating a rise from approximately 625 m/s at 30 bar to 763 m/s at 60 bar (Fig 3). The relationship between nozzle pressure and particle velocity was found to be approximately linear within the investigated pressure range (30–60 bar). Linear regression yielded a high coefficient of determination (R² = 0.98), confirming the strong linearity between applied nozzle pressure and particle velocity. This trend is consistent with the momentum-transfer principle, where higher gas pressures impart greater kinetic energy to the particles, thereby increasing their terminal velocity upon impact.

The slope of the fitted line (3.8 m/s per bar) indicates the sensitivity of velocity to nozzle pressure. Such linear dependence has also been observed in cold spray copper systems, where particle velocities are primarily governed by the drag force balance between gas expansion and particle inertia [17,18].

Elevated particle velocity markedly increases kinetic energy during impact, driving enhanced plastic deformation, adiabatic shear, and metallurgical bonding—mechanisms well-recognised in cold spray deposition theory [9]. This intensification of impact energy underlies the observed increases in lattice microstrain and dislocation density, demonstrating that higher pressure fundamentally enhances microstructural refinement and mechanical interlocking within the sprayed Cu samples.

### 2.2. Surface fracture morphology

**Fig 4:**
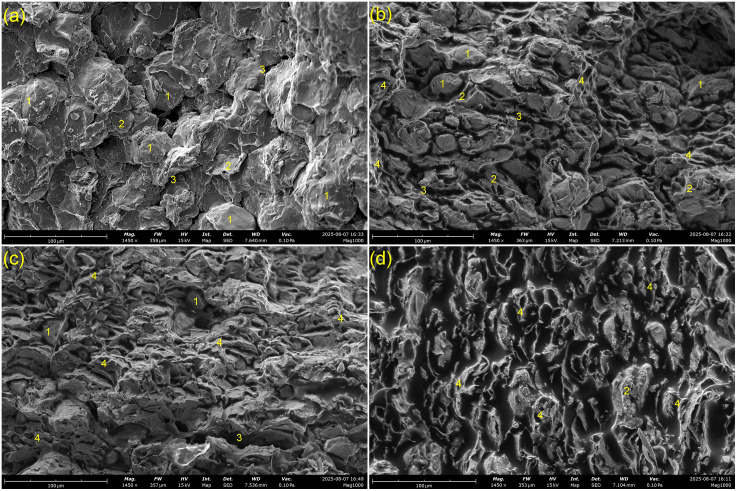
SEM images of fracture surface of samples (a) at 30 bar, (b) at 40 bar, (c) at 50 bar and (c) at 60 bar. The markers show: 1: less deformed particles; 2: dimples caused by plastic deformation (ASI); 3: crack opening sites; 4: bonded area with high deformation.

Fig 4 clearly show the difference in bonding between the particles with different nozzle pressure. At 30 bar, fracture was dominated by inter-particle debonding, with many undeformed particles and weak interfaces, leading to brittle-like fracture and poor elongation. At 40 bar, evidence of stronger bonding and dimple formation emerged, though crack openings were still observed, consistent with moderate ductility. At 50 bar, the surface displayed extensive ductile dimples and strongly bonded regions, correlating with the balance of increased tensile strength and ductility observed in testing. At 60 bar, the fracture surface was dominated by ductile features, with widespread bonding and deep dimples, reflecting excellent plastic deformation capacity.

In cold gas spraying, the bonding of internal interfaces between splats and consequently the resulting deposit properties is governed by high strain-rate plastic deformation and the localised occurrence of adiabatic shear instabilities (ASI) [14]. ASI are initiated when particle impact velocities exceed the material-specific critical velocity [19,20]. Attaining ASI conditions is therefore regarded as a prerequisite for material jetting, oxide rupture, and the establishment of metallurgical bonding. Numerous studies have demonstrated that ASI play a central role in the bonding process [17,21] and that the ASI criterion provides a realistic framework for predicting critical velocities of metals and, by extension, their achievable deposit properties [15,22]. Grujicic et. al [14] suggested the threshold critical velocity for Cu powder was in range of 550–600 m/s. This suggests that 30 bar spray pressure is near/just above critical velocity, thus explains marginal bonding and brittle interfaces while spray pressure of above 40 bar show the significant evolution of microstructure with higher bonding interface.

These observations suggest that fracture mode evolves from brittle interfacial separation at low pressure to fully ductile failure at high pressure.

### 2.3. Electrical conductivity and hardness

**Fig 5:**
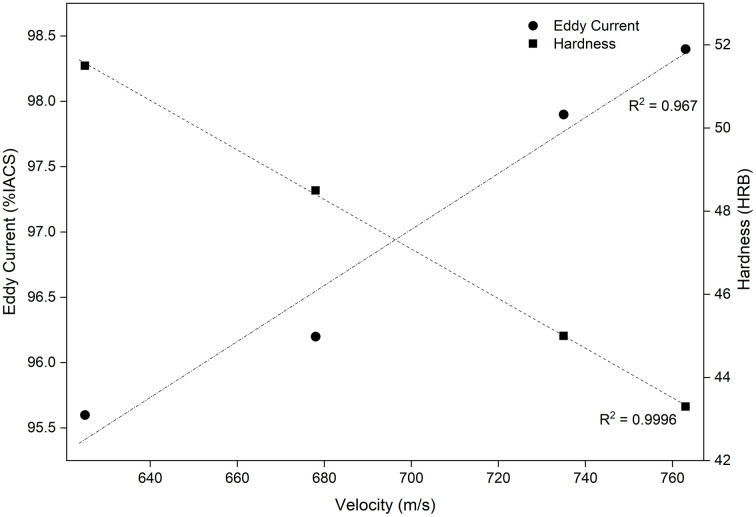
Correlation between electrical conductivity (Eddy Current - %IACS) and hardness (HRB) with particle velocity.

Fig 5 illustrates the variation of hardness and electrical conductivity with particle velocity. Generally, eddy current testing showed that electrical conductivity (expressed in %IACS) improves with increasing cold spray pressure, reaching 98.4% at 60 bar. This enhancement reflects improved interparticle bonding and reduction of detrimental voids or oxide layers at interfaces. In CSAM, electrical transport is more sensitive to such mesoscale discontinuities than to scattering induced by dislocations or grain boundaries. Thus, even though higher pressure elevates dislocation density, the overall improvement in conductivity emphasises the dominant role of densification over lattice scattering effects [9]. These results are in good agreement with fracture morphology observed with SEM.

The improvement of electrical conductivity follows the Drude model:


$$\begin{array}{c} \sigma =(ne^{\text{2}}\tau_{e})/m \end{array}$$

where σ is conductivity, n is electron density, e is electron charge, τₑ is electron relaxation time, and m is electron mass. As pressure increases, improved inter-particle bonding and reduction of oxide barriers increase the effective electron relaxation time (τₑ) by eliminating major scattering centers at particle interfaces, thereby improving conductivity.

Additionally, the increase was particularly pronounced between 40 bar (96.2% IACS) and 50 bar (97.9% IACS), corresponding to a gap of +1.7% IACS, compared to only 0.6% IACS from 30 to 40 bar and 0.5% IACS from 50 to 60 bar. This steep increment implies a critical threshold in particle velocity and impact energy is surpassed at 50 bar, enabling a transition from predominantly mechanical interlocking to enhanced metallurgical bonding. The significant conductivity improvement between 40 and 50 bar suggests that critical particle velocity for copper deposition is reached in this range. Below this threshold, insufficient plastic deformation at particle interfaces leads to incomplete bonding and higher oxide-related porosity [17]. Once this threshold is exceeded, severe plastic deformation causes oxide film rupture and intimate metal–metal contact, improving electrical conductivity [23].

This phenomenon has been reported in previous studies, where eddy current and resistivity measurements strongly correlate with porosity and inter-particle bonding quality [24]. Therefore, the data suggest that 50 bar represents an optimum transition regime, where particle bonding undergoes a qualitative improvement, leading to a denser, more conductive copper sample.

Additionally, a strong negative correlation is observed between hardness and velocity (R² = 0.9996), while conductivity shows a strong positive correlation (R² = 0.967). At lower velocities at 30 bar (625 m/s), sample exhibit higher hardness (52 HRB) but relatively low conductivity (95.6% IACS), reflecting incomplete bonding and porosity. As velocity increases, conductivity rises to 98.4%IACS, demonstrating improved metallurgical bonding and oxide film disruption. Simultaneously, hardness decreases to 43 HRB, consistent with recovery and recrystallisation mechanisms that enable the material to accommodate higher dislocation densities through enhanced plasticity.

These results confirm that increasing spray velocity improves material density and conductivity but reduces hardness, highlighting a strength, ductility and conductivity trade-off that must be optimised depending on application requirements.

### 2.4. XRD Results

#### 2.4.1. XRD pattern.

**Fig 6:**
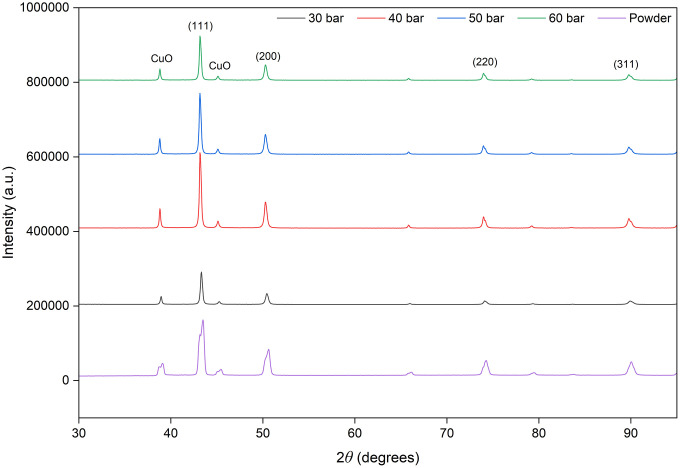
XRD pattern of powder sample and samples printed at different pressure.

Fig 6 reveals the retention of the FCC structure throughout all conditions. The main diffraction peaks corresponding to the (111), (200), (220), and (311) planes remain consistent, indicating no phase transformation induced by the cold spray process. Minor peaks associated with CuO are observed—especially pronounced in the lower-pressure samples—which likely originate from the native oxide layer on the powder surface and residual surface oxidation during powder handling. Notably, the Cu powder exhibits broader diffraction peaks compared to the cold-sprayed samples, which display sharper and more intense peaks. This difference signifies significant microstructural evolution during cold spraying, consistent with severe plastic deformation and possible recovery/recrystallisation-assisted refinement processes and reduced lattice strain. Analysis of the relative peak intensities reveals a pressure-dependent change in crystallographic orientation. The I(111)/I(200) ratio increases systematically from about 2.0 in the powder to about 2.6 at 60 bar, suggesting the development of a weak (111) preferred orientation in sample produced at higher pressure. Such a texture evolution can be attributed to the activation of slip on (111)(110) systems, the dominant deformation mode in FCC Cu [25]. The enhanced (111) texture at higher pressure correlates with improved ductility, as dislocation motion along these close-packed planes provides multiple active slip systems during tensile deformation [26]. Thus, the cold-sprayed copper samples demonstrate increased crystallite size and enhanced crystallinity relative to the initial powder.

The observed broadening of XRD peaks—particularly for (111) and (200)—in cold sprayed samples compared to the initial powder reflects the higher density of microstructural defects introduced by severe plastic deformation. The breadth can be quantified using the Williamson–Hall analysis [27]


$$\begin{array}{c} Bcos\theta =(k\lambda )/D+\text{4}\varepsilon sin\theta \end{array}$$

where B is the full-width at half maximum (FWHM), θ is the Bragg angle, k is the shape factor (0.9), λ is the X-ray wavelength, D is the crystallite size, and ε is the microstrain closely linked to dislocation density. It should be noted that Williamson–Hall analysis provides an estimation of crystallite size and microstrain based on diffraction peak broadening. The technique does not independently distinguish between recovery, polygonisation, recrystallisation, or residual stress contributions. Therefore, the observed trends should be interpreted as indirect indicators of microstructural evolution.

The presence of CuO is most pronounced in the feedstock powder and the 30-bar sample, indicating incomplete disruption of surface oxides at low deposition energy. In contrast, the oxide peaks diminish at higher pressures (≥ 50 bar), suggesting fragmentation or disruption of oxide layers during high-velocity impact and promote clean metallic bonding [17,28]. This observation aligns with the improvement in conductivity values from eddy current testing.

These results highlight the effectiveness of cold spray in modifying the microstructure of Cu powders, producing samples with improved crystal quality and stability without compromising phase integrity.

#### 2.4.2. Crystallise size (nm) and dislocation density (m^-2^).

**Fig 7:**
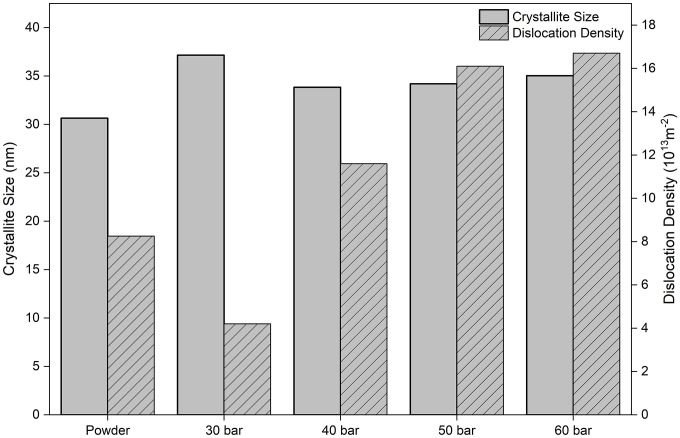
Crystallite size (grey bars) and dislocation density (hatched) of powder sample and samples printed at different pressure at plane (111).

Fig 7 presents the evolution of crystallite size and dislocation density at the (111) plane as a function of cold spray pressure. The feedstock powder exhibited a crystallite size of about 30.65 nm and a dislocation density of about 8.25 x 10^13^ m^-2^. After deposition at 30 bar, the crystallite size increased while dislocation density decreased, indicating insufficient impact energy for effective grain refinement. At this condition, particle velocities of 625 m/s were below the critical velocity required for copper deposition, and only limited interfacial deformation occurred [23].

This can be attributed to both partial annealing effects (arising from frictional heat during particle acceleration) and insufficient impact energy causing only localised deformation without extensive multiplication of dislocations [29–31]

At 40 bar, the critical velocity was exceeded, producing refined crystallites and a sharp rise in dislocation density. This is attributed to oxide film rupture and intense plastic deformation during impact, which generate high defect densities and support strong metallurgical bonding [17]. The increase in particle kinetic energy (with pressure) directly enhances dislocation generation through the activation of Frank-Read sources and shear transfer at particle-particle and particle-substrate interfaces [32]. At 50 bar, crystallite size remained stable while dislocation density plateaued, suggesting that partial dynamic recovery rearranged some dislocations into sub grain structures. At 60 bar, dislocation density reached its highest value at around 16.7 x 10^13^ m^-2^, coupled with further crystallite refinement, reflecting extreme deformation at particle velocities of 763 m/s. Such a defect-rich microstructure, with development of sub grains during the process of recovery, enhances ductility but reduces hardness due to strain accommodation.

Overall, the interplay between crystallite size reduction and dislocation density governs the observed strength and ductility transition. These findings are consistent with the Hall–Petch relationship, where grain refinement enhances strength [26,33], but at very high dislocation densities, dynamic recovery and strain hardening mechanisms improve ductility [25]. The best mechanical performance in cold sprayed Cu samples is achieved in the 50–60 bar range, where refined crystallites and high defect densities provide a balanced combination of strength and ductility.

### 2.5. Mechanical properties

**Fig 8:**
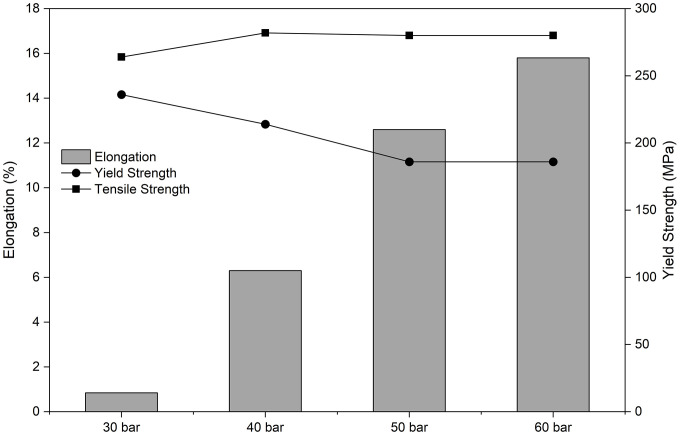
Mechanical strength of samples printed at different pressure.

The tensile behaviour of the cold-sprayed Cu samples exhibited a pronounced dependence on deposition pressure, as shown in Fig 8. At 30 bar, the sample demonstrated extremely low elongation (< 1%) with a relatively high yield strength (YS) (236 MPa) and a lower ultimate tensile strength (UTS) (264 MPa). The poor ductility and brittle-like fracture are attributed to particle velocities being close to, but not significantly above, the critical velocity for copper [14,23]. In this regime, inter-particle bonding remains incomplete, interfaces retain oxide layers, and fracture proceeds primarily along weak particle boundaries, as confirmed by the SEM fracture surfaces.

The paradoxical relationship between increased dislocation density and enhanced ductility in cold-sprayed materials differs fundamentally from conventional work-hardened metals. While work hardening typically exhausts ductility through dislocation accumulation, cold spray generates a unique heterogeneous microstructure where localised high-strain regions coexist with less-deformed zones, enabling progressive strain accommodation through multiple mechanisms. This phenomenon parallels recent findings in nano twinned copper and gradient nanostructured materials, where spatial separation of strengthening and toughening mechanisms enables simultaneous enhancement of both properties [34].

When the spray pressure increased to 40 bar, particle velocities (678 m/s) well exceeded the critical threshold, leading to substantial improvements in bonding and plastic deformation at particle interfaces. This resulted in a significant increase in elongation (6.3%) and a modest reduction in yield strength (214 MPa). At the microstructural level, the sharp rise in dislocation density (Fig 7) and the reduction in crystallite size indicate that severe plastic deformation is consistent with recovery/recrystallisation-assisted refinement mechanisms (cDRX), forming refined grains at particle boundaries [35,36].

The observed strengthening can be quantified through the Taylor hardening relationship [37]:


$$\begin{array}{c} \tau =\tau_{\text{0}}+\alpha Gb\rho \end{array}$$

where τ is the critical resolved shear stress, τ₀ is the friction stress, α is the forest hardening coefficient (~0.3 for copper), G is the shear modulus (48 GPa for copper), b is the Burgers vector (2.556 × 10 ⁻ ¹⁰ m), and ρ is the dislocation density. However, the simultaneous improvement in ductility indicates that enhanced inter-particle bonding and DRX mechanisms enable the material to accommodate the increased dislocation density without brittleness [38].

Such DRX-assisted grain subdivision enhanced ductility by providing new dislocation sources and reducing interfacial stresses, while simultaneously strengthening inter-particle cohesion.

Specifically, the DRX-refined regions act as strain accommodation sites through: (i) enhanced dislocation generation from newly formed grain boundaries serving as Frank-Read sources, (ii) activation of multiple <110 > slip systems in differently oriented grains—the dominant slip mode in FCC copper, and (iii) grain boundary sliding at ultrafine grain interfaces. The measured dislocation density from XRD represents statistically stored dislocations (SSDs) within crystallite domains, while geometrically necessary dislocations (GNDs) at particle boundaries—not captured by XRD—provide additional plasticity through interface-mediated deformation.

Adiabatic shear is generally accompanied by increase in temperature resulting in DRX. At 50 bar, elongation continued to rise (12.6%), while YS further decreased (186 MPa), despite the dislocation density increasing to ~14 × 10¹³ m ⁻ ². This apparent contradiction with classical hardening theory results from multiple microstructural factors: (i) elimination of stress concentrators at particle interfaces through improved metallurgical bonding (as evidenced by the conductivity increase from 96.2% to 97.9% IACS), (ii) formation of dislocation cell structures that facilitate plastic deformation, and (iii) dynamic recovery processes that rearrange dislocations into less harmful configurations.

The slight stabilisation of dislocation density increase compared to 40 bar suggests the onset of a balance between dislocation generation and recovery, where tangled dislocations rearrange into subgrain boundaries, enabling plastic deformation [25,26]. Despite this, UTS remained stable (280 MPa), highlighting that strain-hardening capacity was preserved due to refined grains and DRX-produced high-angle boundaries, which resist dislocation motion (Fig 7).

At 60 bar, elongation peaked (15.8%), while YS stabilised (186 MPa) and UTS remained nearly unchanged (280 MPa) resulting in enhanced strain hardening rate. The fully ductile fracture surface, dominated by extensive dimples and highly deformed bonded regions, confirms bulk-like plasticity at this stage.

The high dislocation density enables ductility through a dynamic recovery-recrystallisation balance: while dislocations multiply during deformation following the Orowan relation γ̇ = ρbv (where γ̇ is plastic shear strain rate, b is Burgers vector, and v is dislocation velocity), concurrent recovery processes (cross-slip, climb) and continuous DRX prevent strain [39]. The extremely high strain rates during particle impact (10^6^−10^8^ s^-1^) modify dislocation dynamics significantly—the high applied stresses reduce dislocation loop aspect ratios and increase dislocation velocities, promoting more homogeneous deformation rather than localised pile-ups characteristic of quasi-static loading. This creates a self-regulating microstructure that maintains work hardening capability (stable UTS of 280 MPa) while accommodating large plastic strains, similar to mechanisms observed in deformed and partitioned steels with high dislocation densities [40]. Additionally, the significant improvement in inter-particle continuity inferred from conductivity behaviour (implied by conductivity approaching 98.4% IACS) removes premature failure sites, allowing the dislocation-mediated deformation mechanisms to fully operate.

**Fig 9:**
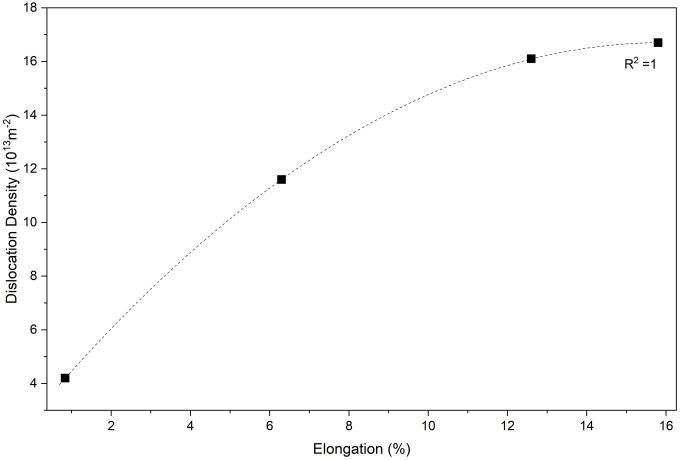
Correlation between elongation and dislocation density.

The relationship between dislocation density (ρ) and tensile elongation is presented in Fig 9. A clear positive correlation is observed, where elongation increases from <1% at 4 x 10^13^ m^-2^ to 16% at 16.7 x 10^13^ m^-2^.

This trend, which contradicts classical strengthening theory, demonstrates that ductility in cold-sprayed Cu is governed not by dislocation density alone but by the synergistic evolution of multiple microstructural features. The progressive improvement in inter-particle bonding quality with increasing pressure transforms the material from a weakly-bonded, porous structure to a fully dense material capable of accommodating high dislocation densities through plastic deformation.

The enhanced ductility arises from the synergistic interaction of: (i) SSDs providing strain hardening according to the Kocks-Mecking framework [41], (ii) GNDs accommodating strain gradients at particle boundaries as described by the Nye-Kröner theory, (iii) progressive reduction in porosity enabling load transfer across the microstructure, (iv) fragmentation and dispersion of oxide inclusions removing brittle failure sites, and (v) DRX-induced grain refinement creating multiple deformation pathways. The strong correlation between elongation and SSD density suggests that dislocation density serves as a proxy indicator for the overall microstructural evolution, including bonding improvement and porosity reduction, rather than being the sole governing factor.

At low pressures (30 bar), insufficient density results in limited plastic deformation and brittle fracture along particle interfaces. At intermediate pressures (40–50 bar), the higher dislocation density, supported by continuous dynamic recrystallisation (cDRX), enhances slip activity on (111) planes, leading to substantial gains in ductility. At 60 bar, elongation saturates as dynamic recovery through sub grain refinement stabilises the microstructure, even as dislocation density remains high. This saturation phenomenon has been observed in SPD-processed copper, where a balance between defect accumulation and dynamic recovery establishes a steady-state microstructure. Future work should quantify the individual contributions of porosity reduction, GND evolution measured via HR-EBSD, and true grain size distribution via EBSD to fully deconvolute the ductility enhancement mechanisms.

Residual stress may also contribute to the observed tensile behaviour and fracture characteristics. Cold spray deposition is known to generate heterogeneous residual stress states due to severe localised deformation and thermal gradients during particle impact. Since residual stress was not measured in the present study, its contribution cannot be quantitatively separated from the observed microstructural effects. Future work incorporating XRD residual stress analysis or neutron diffraction would provide further insight into stress–microstructure interactions.

In brief, these results demonstrate that deposition pressure governs tensile properties through its control of particle velocity, bonding quality, and microstructure. At low pressures, sample exhibit brittle fracture due to insufficient deformation. At intermediate pressures (40–50 bar), the balance of dislocation multiplication and recovery optimises strength and ductility. At high pressures (60 bar), ductility is maximised due to severe plastic deformation and extensive slip activity, while strength stabilises due to preserved strain-hardening mechanisms.

## 3. Limitation and future work

Although this study establishes a correlation between spray pressure, particle velocity, microstructural evolution, and mechanical/electrical performance, several limitations remain. The present work relied primarily on XRD peak broadening and SEM observations to interpret microstructural evolution. Direct validation of recrystallisation behaviour using EBSD or TEM was not performed. Similarly, porosity, oxide distribution, and inter-splat bonding were not quantitatively measured. Residual stress effects were also not investigated. Furthermore, only spray pressure was varied, while other coupled parameters such as gas temperature, stand-off distance, feed rate, and nozzle geometry were held constant.

Future work should incorporate EBSD, TEM, residual stress analysis, porosity quantification, and chemical surface analysis to further establish the mechanisms governing bonding and ductility evolution in cold-sprayed copper.

## 4. Conclusion

In this study, the effect of cold spray deposition pressure on the microstructure and mechanical/electrical performance of copper coatings was systematically investigated through XRD line profile analysis, hardness and eddy-current measurements, tensile testing, and SEM fracture morphology observations. The following conclusions can be drawn:

- Critical velocity governs the transition in bonding mechanism. At 30 bar (particle velocity 625 m/s), deposition occurs near the critical velocity for Cu, resulting in weak inter-particle bonding, intact oxide layers, and brittle fracture with negligible ductility. Increasing the pressure to ≥40 bar (velocity ≥678 m/s) surpasses the critical threshold, enabling oxide rupture, plastic deformation, and metallurgical bonding between particles.
- Microstructural refinement consistent with recovery/recrystallisation-assisted mechanisms was observed. XRD analysis revealed progressive grain refinement (crystallite size decreasing to 33–35 nm) and increased dislocation density (up to 16.7 x 10^13^ m^-2^ at 60 bar), indicating continuous DRX during high-strain, high-strain-rate impacts. This refinement enhances dislocation mobility and provides new slip sources, underpinning the improved ductility of coatings deposited at higher pressures.
- Strength, ductility and conductivity trade-off is pressure dependent. Hardness decreased almost linearly with particle velocity (52–43 HRB), while electrical conductivity increased (95.6% to 98.4% IACS), reflecting simultaneous recovery/DRX softening and densification of the coating. Tensile tests confirmed a strength–ductility transition: yield strength decreased from 236 to 186 MPa, elongation rose from 0.8% to 15.8%, and ultimate tensile strength stabilised at 280 MPa once sufficient bonding was achieved.
- Fracture mode evolves from interfacial to substantially improved ductile behaviour approaching bulk copper response. SEM fracture analysis revealed a transition from brittle interfacial separation (30 bar) to mixed fracture with partial dimpling (40 bar), to fully ductile dimpled fracture at 50–60 bar. This evolution is consistent with microstructural evidence of DRX and improved bonding quality.
- Optimal processing window (50–60 bar). Within this pressure range, coatings achieve a balanced property profile: high electrical conductivity, refined microstructure with significant DRX, stable tensile strength, and excellent ductility. This highlights the importance of tuning deposition pressure to surpass critical velocity while promoting controlled DRX for multifunctional copper components.
- Although higher spray pressures increase gas consumption and processing cost, the identified 50–60 bar processing window provides a favourable balance between conductivity, ductility, and bonding quality for industrial CSAM applications

## References

[1] Papyrin A. Cold spray technology. Elsevier. 2006.

[2] Moridi A, et al. Cold spray coating: review of material systems and future perspectives. Surf Eng. 2014;30(6):369–95. doi: 10.1179/1743294414Y.0000000270

[3] Blomgren M, Gunnarsson S. Cold spraying as a repair method for crankshaft journals. 2023. http://hdl.handle.net/20.500.12380/306242

[4] Prashar G, Vasudev H. A comprehensive review on sustainable cold spray additive manufacturing: State of the art, challenges and future challenges. J Clean Prod. 2021;310:127606. doi: 10.1016/j.jclepro.2021.127606

[5] Vaz R, Garfias A, Albaladejo V, Sanchez J, Cano I. A Review of Advances in Cold Spray Additive Manufacturing. Coatings. 2023;13(2):267. doi: 10.3390/coatings13020267

[6] Kumar M, Singh J, Uppal AS. Improvement in corrosion resistance of AISI 316L stainless steel weld cladding using GTA remelting technique. Materials Today: Proceedings. 2022;65:3224–8. doi: 10.1016/j.matpr.2022.05.377

[7] Kumar M, et al. Recent progress in 5D printing: processes, materials, applications, and future trends. Advanced Materials Technologies. 2026;:e01337. doi: 10.1002/admt.202501337

[8] Villafuerte J. Modern cold spray: materials, process, and applications. 2015: Springer. doi: 10.1007/978-3-319-16772-5

[9] Rokni MR, Nutt SR, Widener CA, Champagne VK, Hrabe RH. Review of Relationship Between Particle Deformation, Coating Microstructure, and Properties in High-Pressure Cold Spray. J Therm Spray Tech. 2017;26(6):1308–55. doi: 10.1007/s11666-017-0575-0

[10] Yin S, et al. Effect of injection pressure on particle acceleration, dispersion and deposition in cold spray. Comput Mater Sci. 2014;90:7–15. doi: 10.1016/j.commatsci.2014.03.055

[11] Gärtner F, Stoltenhoff T, Schmidt T, Kreye H. The Cold Spray Process and Its Potential for Industrial Applications. Journal of Thermal Spray Technology. 2006;15(2):223–32. doi: 10.1361/105996306x108110

[12] Champagne VK, Helfritch D, Hassani M. Advances in Cold Spray: A Coating Deposition and Additive Manufacturing Process. Helfritch MHD, Champagne VK, editors. Woodhead Publishing. 2023. doi: 10.1016/C2018-0-00432-6

[13] Sudharshan Phani P, Srinivasa Rao D, Joshi SV, Sundararajan G. Effect of Process Parameters and Heat Treatments on Properties of Cold Sprayed Copper Coatings. J Therm Spray Tech. 2007;16(3):425–34. doi: 10.1007/s11666-007-9048-1

[14] Grujicic M, et al. Adiabatic shear instability based mechanism for particles/substrate bonding in the cold-gas dynamic-spray process. Mater Des. 2004;25(8):681–8. doi: 10.1016/j.matdes.2004.03.008

[15] Assadi H, Gärtner F, Klassen T, Kreye H. Comment on ‘Adiabatic shear instability is not necessary for adhesion in cold spray’. Scripta Materialia. 2019;162:512–4. doi: 10.1016/j.scriptamat.2018.10.036

[16] Ji G, et al. Effect of friction stir spot processing on microstructure and mechanical properties of cold-sprayed Al coating on Ti substrate. Surf Coat Tech. 2021;421:127352. doi: 10.1016/j.surfcoat.2021.127352

[17] Assadi H, Gärtner F, Stoltenhoff T, Kreye H. Bonding mechanism in cold gas spraying. Acta Materialia. 2003;51(15):4379–94. doi: 10.1016/s1359-6454(03)00274-x

[18] Shushpanov A. Gas-dynamic spraying method for applying a coating. U. States. 1994. https://patentimages.storage.googleapis.com/92/16/7f/93e032d9041894/US5302414.pdf

[19] Schmidt T, Gärtner F, Assadi H, Kreye H. Development of a generalized parameter window for cold spray deposition. Acta Materialia. 2006;54(3):729–42. doi: 10.1016/j.actamat.2005.10.005

[20] Schmidt T, Assadi H, Gärtner F, Richter H, Stoltenhoff T, Kreye H, et al. From Particle Acceleration to Impact and Bonding in Cold Spraying. J Therm Spray Tech. 2009;18(5–6). doi: 10.1007/s11666-009-9357-7

[21] Huang C, List A, Shen J, Fu B, Yin S, Chen T, et al. Tailoring powder strengths for enhanced quality of cold sprayed Al6061 deposits. Materials & Design. 2022;215:110494. doi: 10.1016/j.matdes.2022.110494

[22] "Lima R, et al. Mechanical properties of cold-sprayed Ti-6Al-4V coatings. In: Thermal Spray 2013: Proceedings from the International Thermal Spray Conference, 2013. 10.31399/asm.cp.itsc2013p0155

[23] Zhang C, et al. Critical velocity and deposition efficiency in cold spray: A reduced-order model and experimental validation. J Manuf Process. 2025;134:547–57. doi: 10.1016/j.jmapro.2024.12.077

[24] Itoh Y, Suyama S, Fukanuma H. Thermal and electrical properties of copper coatings produced by cold spraying. J Soc Mater Sci Jpn. 2010;59(2):143–8. doi: 10.2472/jsms.59.143

[25] Humphreys FJ, Hatherly M. Recrystallization and related annealing phenomena. Elsevier. 2012. doi: 10.1016/B978-0-08-044164-1.X5000-2

[26] Meyers MA, Chawla KK. Mechanical behavior of materials. 2nd ed. Cambridge University Press. 2008. doi: 10.1017/CBO9780511810947

[27] Warren BE. X-Ray Diffraction. Dover Publications. 1990.

[28] Li C-J, Li W-Y, Liao H. Examination of the Critical Velocity for Deposition of Particles in Cold Spraying. Journal of Thermal Spray Technology. 2006;15(2):212–22. doi: 10.1361/105996306x108093

[29] Seo D, Ogawa K, Sakaguchi K, Miyamoto N, Tsuzuki Y. Parameter study influencing thermal conductivity of annealed pure copper coatings deposited by selective cold spray processes. Surface and Coatings Technology. 2012;206(8–9):2316–24. doi: 10.1016/j.surfcoat.2011.10.010

[30] Devi GN, Kumar S, Mangalarapu TB, Vinay G, Chavan NM, Gopal AV. Assessing critical process condition for bonding in cold spraying. Surface and Coatings Technology. 2023;470:129839. doi: 10.1016/j.surfcoat.2023.129839

[31] Lucas TJ, Schuh CA. A single particle impact study on the effects of substrate strength and ductility on adhesion in cold spray. International Journal of Mechanical Sciences. 2025;303:110676. doi: 10.1016/j.ijmecsci.2025.110676

[32] Xu S, Xiong L, Chen Y, McDowell DL. An analysis of key characteristics of the Frank-Read source process in FCC metals. Journal of the Mechanics and Physics of Solids. 2016;96:460–76. doi: 10.1016/j.jmps.2016.08.002

[33] Hosford WF. Mechanical behavior of materials. Cambridge University Press. 2010. doi: 10.1017/CBO9780511810923

[34] Lu L, et al. Ultrahigh strength and high electrical conductivity in copper. Science. 2004;304(5669):422–6. doi: 10.1126/science.1092905 15031435

[35] Zou Y, et al. Dynamic recrystallization in the particle/particle interfacial region of cold-sprayed nickel coating: Electron backscatter diffraction characterization. Scr Mater. 2009;61(9):899–902. doi: 10.1016/j.scriptamat.2009.07.020

[36] Kafle A, Lu S, Silwal R, Zhu W. A Review on Material Dynamics in Cold Spray Additive Manufacturing: Bonding, Stress, and Structural Evolution in Metals. Metals. 2025;15(2):187. doi: 10.3390/met15020187

[37] Kocks UF. The relation between polycrystal deformation and single-crystal deformation. Metall Trans. 1970;1(5):1121–43. doi: 10.1007/bf02900224

[38] Vinay G, Halder S, Kant R, Singh H. Examining the contribution of tamping effect on inter-splat bonding during cold spray. Materials Science and Engineering: A. 2024;893:146112. doi: 10.1016/j.msea.2024.146112

[39] Ward S. The effects of microstructure and nanostructure upon dynamic ductile fracture. 2022. doi: 10.17863/CAM.90275

[40] He BB, Hu B, Yen HW, Cheng GJ, Wang ZK, Luo HW, et al. High dislocation density-induced large ductility in deformed and partitioned steels. Science. 2017;357(6355):1029–32. doi: 10.1126/science.aan0177 28839008

[41] Kocks UF, Mecking H. Physics and phenomenology of strain hardening: the FCC case. Progress in Materials Science. 2003;48(3):171–273. doi: 10.1016/s0079-6425(02)00003-8

