# Supplementary material for: Optimising Cold Spray Additive Manufacturing: Pressure-driven enhancement of mechanical performance in copper deposits
Source: PLoS One. 2026 Jul 2;21(7):e0353072. doi: 10.1371/journal.pone.0353072 (PMC13327120; doi:10.1371/journal.pone.0353072)
Supplement: Attachment — Submitted filename: Response to Reviewers.docx [file pone.0353072.s002.docx]

# Response to Reviewers

**Manuscript Title:** Optimizing Cold Spray Additive Manufacturing: Pressure-Driven Enhancement of Mechanical Performance
**Manuscript ID:** [PONE-D-26-18004]

We sincerely thank the reviewers for their constructive and insightful comments. These have significantly improved the clarity, rigor, and scope of the manuscript. All comments have been carefully addressed, and the manuscript has been revised accordingly. A detailed response is provided below.

**Reviewer #1**: **- The authors must explicitly clarify how this work advances beyond existing studies—particularly whether the contribution lies in quantitative correlation, LightSPEE3D-specific processing, or integrated mechanical–electrical optimization.**

**Response:**

We thank the reviewer for this important comment. The novelty and contribution of the study have been clarified in the revised Introduction and Abstract. The manuscript now explicitly highlights that the present work establishes a process, structure and property relationship linking particle velocity, XRD-derived microstructural evolution, electrical conductivity, and tensile behaviour within a LightSPEE3D CSAM system. In addition, the study identifies a transition regime between 40–50 bar associated with significant changes in bonding behaviour and multifunctional performance.

**- The conclusions strongly rely on particle velocity values and their relation to critical velocity; however, there is no clear description of how velocity was measured or validated**

**Response:**

The velocity measurement was described in section 2.3 “Velocity of powder particles out of nozzle measurement”. It has detailed description of the Oseir HiWatch CS2 particle velocimetry system, including laser pulse interval, image acquisition conditions, particle tracking methodology, and averaging procedure.

**- Porosity reduction and bonding improvement lack quantitative validation; no measurements of porosity, inter-splat bonding, or oxide thickness and distribution are provided.**

**Response:**

We agree that direct porosity and oxide quantification would further strengthen the study. In the revised manuscript, statements regarding porosity reduction and densification have been moderated and are now presented as inferred trends based on conductivity behaviour and fracture morphology rather than direct measurements. In addition, a dedicated “Limitations and Future Work” section has been added to acknowledge the absence of quantitative porosity measurements, interfacial oxide characterization, and inter-splat bonding analysis. Future work involving image analysis, X-ray CT, and high-resolution microscopy has been proposed.

**- The manuscript does not address residual stress, which is a key factor in interpreting tensile and fracture behavior.**

**Response:**

We thank the reviewer for this valuable observation. A discussion regarding the potential influence of residual stress on tensile behaviour and fracture characteristics has been added to the revised Discussion section. The manuscript now explicitly acknowledges that residual stress was not measured and that future work involving XRD residual stress analysis or neutron diffraction would provide further insight into stress–microstructure interactions.

**- Strength–ductility trade-off is inadequately explained: lacks dislocation density validation, no strengthening models applied, and ultimate tensile strength stabilization remains mechanistically unclear.**

**Response:**

The Discussion section has been substantially revised to improve the mechanistic interpretation of the strength–ductility relationship. The revised manuscript now incorporates Hall–Petch strengthening concepts, hardening, and recovery/recrystallisation-assisted deformation mechanisms to explain the observed reduction in hardness and yield strength alongside improved ductility. We have also moderated several mechanistic claims to avoid over-interpretation beyond the available experimental evidence.

**- The study focuses only on spray pressure, but other important factors like gas temperature, powder size, stand-off distance, and nozzle design are overlooked, limiting broader applicability.**

**Response:**

We thank the reviewer for this valuable comment. The revised manuscript has been updated to clarify that gas temperature and stand-off distance were controlled during deposition and are now explicitly reported in the Experimental Procedure section (gas temperature: 750 °C; stand-off distance: 20 mm and federate of 60g/s).

The present study intentionally focused on spray pressure as the primary independent variable in order to establish a clearer process–structure–property relationship between pressure, particle velocity, microstructural evolution, and multifunctional performance in cold-sprayed copper. Powder size distribution was described in the manuscript.

We acknowledge that other parameters including nozzle geometry also influence particle acceleration and bonding behaviour. However, several of these parameters are associated with proprietary industrial system configurations within the LightSPEE3D platform and therefore could not be fully disclosed. To address this limitation, the manuscript now explicitly states that the identified processing window is specific to the operating conditions of the present system and should not be interpreted as universally transferable to all cold spray systems.

Additional discussion regarding the influence of coupled process parameters has also been incorporated into the Discussion and Limitations sections of the revised manuscript.

- **In introduction section these references may be helpful:
\# Recent Progress in 5D Printing: Processes, Materials, Applications, and Future Trends
#Improvement in corrosion resistance of AISI 316L stainless steel weld cladding using GTA remelting technique
\# Sensitization studies on low cycle fatigue and cryogenic impact behavior of AISI 304 SS weldments**

**Response:**

We have added those references into the Introduction section of the manuscript to strengthen the novelty of the manuscript.

**Reviewer #2:**

1. **The manuscript addresses an industrially relevant topic, but the novelty is moderate because the influence of spray pressure on cold-sprayed copper has been previously reported, and the present study should more clearly define its unique mechanistic contribution beyond existing pressure–property studies.**

**Response:**

We appreciate this comment and have revised the Introduction to better clarify the novelty of the work. While previous studies have investigated spray pressure effects independently, the present study focuses on establishing an integrated correlation between particle velocity, XRD-derived microstructural evolution, electrical conductivity, and tensile behaviour in a CS system. The identification of a transition regime between 40–50 bar has also been emphasized.

2. **The study focuses only on spray pressure, while other strongly coupled deposition parameters (gas temperature, standoff distance, feed rate, nozzle traverse speed) are held constant and insufficiently discussed, limiting the broader applicability of the conclusions.**

**Response:**

The revised manuscript now explicitly acknowledges that the present study isolates spray pressure while holding other process parameters constant in order to establish a clearer process–property relationship. Additional discussion regarding the influence of gas temperature, feed rate, stand-off distance, and nozzle geometry has been added.

3. **The justification for selecting only four pressure levels (30, 40, 50, and 60 bar) is not adequately explained, and intermediate values could strengthen the identification of the proposed optimum processing window**.

**Response:**

The manuscript has been revised to clarify that the selected pressure range (30–60 bar) was intentionally chosen to span conditions near, below, and above the expected critical velocity regime for copper deposition. This enabled identification of the transition from weak bonding behaviour to improved metallurgical bonding.

4. **The manuscript repeatedly attributes improved ductility to continuous dynamic recrystallization (cDRX), but this central claim is inferred indirectly from XRD broadening and SEM rather than being directly validated using EBSD or TEM.**

**Response:**

We agree and have carefully moderated the discussion throughout the manuscript. Statements previously implying direct confirmation of cDRX have been revised to indicate that the observed behaviour is “consistent with” or “suggestive of” recovery/recrystallisation-assisted refinement mechanisms. The revised manuscript now explicitly states that EBSD or TEM would be required for direct validation for future suggestion.

5. **The use of Williamson–Hall analysis alone to estimate crystallite size and dislocation density is insufficient for a strong mechanistic claim, especially in heavily deformed copper where instrumental broadening and residual stress can significantly affect peak width.**

**Response:**

We acknowledge this limitation. Additional discussion has been added clarifying that Williamson–Hall analysis provides an estimation of crystallite size and microstrain based on peak broadening and does not independently distinguish between recrystallisation, recovery, or residual stress contributions.

6. **The discussion of dynamic recrystallization is overstated, as no direct grain orientation maps or recrystallized fraction measurements are presented to distinguish recovery, polygonization, and true recrystallization.**

**Response:**

The Discussion and Conclusion sections have been revised to reduce overstatement regarding dynamic recrystallisation. Mechanistic interpretations are now presented more cautiously and framed as indirect evidence consistent with severe plastic deformation and recovery/recrystallisation-assisted refinement processes.

7. **The hardness and conductivity trends are interesting, but the manuscript does not adequately explain the apparent trade-off between reduced hardness and improved tensile ductility using established strengthening models.**

**Response:**

We have expanded the mechanistic discussion using Hall–Petch strengthening concepts, Taylor hardening, and recovery-assisted strain accommodation mechanisms to better explain the simultaneous reduction in hardness and improvement in ductility/conductivity.

8. **The conductivity values are reported close to bulk copper (up to 98.4% IACS), but the manuscript does not sufficiently discuss possible measurement uncertainty, surface effects, or anisotropy in eddy current testing.**

**Response:**

The Materials and Methods section has been expanded to include calibration procedure, testing frequency, repeated measurements, and testing methodology using the Fischer Sigmascope SMP350. Additional discussion has also been added to the possibility of uncertainty associated with surface condition and anisotropy.

9. **The claim of “bulk-like plasticity” is somewhat overstated, as the tensile strength remains significantly lower than wrought copper and should be discussed more cautiously.**

**Response:**

We agree and have revised the wording throughout the manuscript. The phrase “bulk-like plasticity” has been replaced with more cautious terminology such as “substantially improved ductility approaching bulk copper behaviour.”

10. **The fracture analysis is qualitatively described, but the SEM fractography would benefit from quantitative dimple size analysis or fracture surface area comparison to support the ductile transition claim.**

**Response:**

We acknowledge this limitation and have added a statement in the revised manuscript noting that quantitative dimple size analysis would further strengthen the interpretation of fracture behaviour and should be considered in future work.

11. **The manuscript lacks porosity quantification, despite porosity being repeatedly invoked as a major reason for conductivity and ductility changes.**

**Response:**

We agree and have moderated all direct claims regarding porosity reduction. The revised manuscript now presents densification and improved bonding as inferred trends supported by conductivity and fracture morphology observations rather than direct measurements. This limitation is explicitly acknowledged.

12. **The role of oxide rupture is central to the bonding mechanism, yet no chemical surface analysis (e.g., XPS or EDS mapping) is provided to directly support oxide disruption or reduction with pressure.**

**Response:**

The manuscript has been revised to reduce overstatement regarding oxide rupture. The discussion now refers more cautiously to “possible oxide fragmentation/disruption” inferred from conductivity improvement and XRD trends. The need for future EDS/XPS characterization has also been acknowledged.

13. **Residual stress, which is highly relevant in cold spray deposits and can strongly influence ductility and cracking, is not measured or discussed.**

**Response:**

A dedicated discussion regarding the possible influence of residual stress has now been added to the revised manuscript, together with acknowledgment of this limitation and recommendations for future investigation.

14. **The particle velocity measurements are useful, but the manuscript does not sufficiently discuss velocity distribution spread, particle size dependence, or deposition efficiency, all of which are important for interpreting process–structure relationships.**

**Response:**

The revised manuscript now clarifies that reported particle velocities represent mean values and that actual deposition involves a distribution influenced by particle size and gas dynamics. We acknowledge that deposition efficiency and velocity spread were not quantitatively analysed in the present work.

15. **The industrial significance is promising, but the manuscript would be stronger if the authors discussed how the identified 50–60 bar processing window translates into cost, energy consumption, and scalability in industrial CSAM.**

**Response:**

The revised Discussion section has been expanded to discuss the industrial implications of the identified 50–60 bar processing window, including the balance between improved deposit performance and increased gas pressure/energy requirements in practical CSAM applications.

# Response to Academic Editor

We sincerely thank the Editor for the constructive and insightful comments and for the opportunity to revise our manuscript entitled “Optimising Cold Spray Additive Manufacturing: Pressure-Driven Enhancement of Mechanical Performance in Copper Deposits”.

As requested, we have updated the manuscript as suggested to meet PLOS ONE’s style requirements. We have updated the data availability link for peer review. The data should be published and freely accessible if the manuscript is accepted for publication. We have amended the manuscript submission data to include author Thi Thuy Tien Tran. Figure 3 has been referred to in the manuscript.

We also updated the manuscript for both the Funding Statement and Competing Interests Statement to clarify the commercial affiliation associated with SPEE3D and the role of the funder in this study.

The revised Funding Statement is as follows:

“This research was supported by the Department of Education Australia through the Department of Education’s National Industry PhD Program (NIPhD 35017). The views expressed herein are those of the authors and are not necessarily those of the Australian Government or the Department of Education. There was no additional external funding received for this study. This work also received support from SPEE3D in the form of salaries for authors SC, TT, AD, MH, and RR, as well as access to materials and manufacturing facilities, but did not have any additional role in the study design, data collection and analysis, decision to publish, or preparation of the manuscript. The specific roles of these authors are articulated in the ‘author contributions’ section. The study was conducted collaboratively between Charles Darwin University and SPEE3D.”

The revised Competing Interests Statement is as follows:

“The authors have read the journal’s policy and have the following competing interests: Steven Camilleri, Tien Tran, Andrew Duguid, Matthew Harbidge, and Riyan Rashid are employees of SPEE3D. SPEE3D provided support in the form of salaries, materials, and access to manufacturing facilities. This does not alter our adherence to PLOS ONE policies on sharing data and materials.”
