# Supplementary material for: Optimising Cold Spray Additive Manufacturing: Pressure-driven enhancement of mechanical performance in copper deposits
Source: PLoS One. 2026 Jul 2;21(7):e0353072. doi: 10.1371/journal.pone.0353072 (PMC13327120; doi:10.1371/journal.pone.0353072)
Supplement: Peer review history

# Peer review: Decision Letter 0

**DOI:** 10.1371/journal.pone.0353072.r001

**Authors:** Gaurav Arora

4 May 2026

PONE-D-26-18004

Optimising Cold Spray Additive Manufacturing: Pressure-Driven Enhancement of Mechanical Performance in Copper Deposits

PLOS One

Dear Dr. Tran,

Thank you for submitting your manuscript to PLOS ONE. After careful consideration, we feel that it has merit but does not fully meet PLOS ONE’s publication criteria as it currently stands. Therefore, we invite you to submit a revised version of the manuscript that addresses the points raised during the review process.

==============================

**ACADEMIC EDITOR:**  The manuscript needs the revisions as suggested by the reviewers.

==============================

Please submit your revised manuscript by Jun 18 2026 11:59PM. If you will need more time than this to complete your revisions, please reply to this message or contact the journal office at plosone@plos.org. When you're ready to submit your revision, log on to https://www.editorialmanager.com/pone/ and select the 'Submissions Needing Revision' folder to locate your manuscript file.

Please include the following items when submitting your revised manuscript:

- A letter that responds to each point raised by the academic editor and reviewer(s). You should upload this letter as a separate file labeled 'Response to Reviewers'.
- A marked-up copy of your manuscript that highlights changes made to the original version. You should upload this as a separate file labeled 'Revised Manuscript with Track Changes'.
- An unmarked version of your revised paper without tracked changes. You should upload this as a separate file labeled 'Manuscript'.

If you would like to make changes to your financial disclosure, please include your updated statement in your cover letter. Guidelines for resubmitting your figure files are available below the reviewer comments at the end of this letter.

If applicable, we recommend that you deposit your laboratory protocols in protocols.io to enhance the reproducibility of your results. Protocols.io assigns your protocol its own identifier (DOI) so that it can be cited independently in the future. For instructions see: https://journals.plos.org/plosone/s/submission-guidelines#loc-laboratory-protocols. Additionally, PLOS ONE offers an option for publishing peer-reviewed Lab Protocol articles, which describe protocols hosted on protocols.io. Read more information on sharing protocols at https://plos.org/protocols?utm_medium=editorial-email&utm_source=authorletters&utm_campaign=protocols.

As the corresponding author, your ORCID iD is verified in the submission system and will appear in the published article. PLOS supports the use of ORCID, and we encourage all coauthors to register for an ORCID iD and use it as well. Please encourage your coauthors to verify their ORCID iD within the submission system before final acceptance, as unverified ORCID iDs will not appear in the published article. *Only*  the individual author can complete the verification step; PLOS staff *cannot*  verify ORCID iDs on behalf of authors.

We look forward to receiving your revised manuscript.

Kind regards,

Gaurav Arora, Ph.D

Academic Editor

PLOS One

Journal Requirements:

When submitting your revision, we need you to address these additional requirements.

1. Please ensure that your manuscript meets PLOS ONE's style requirements, including those for file naming. The PLOS ONE style templates can be found at https://journals.plos.org/plosone/s/file?id=wjVg/PLOSOne_formatting_sample_main_body.pdf and https://journals.plos.org/plosone/s/file?id=ba62/PLOSOne_formatting_sample_title_authors_affiliations.pdf

2. Thank you for stating the following in the Acknowledgments Section of your manuscript:

[The authors would like to acknowledge the support of the technical staff at Charles Darwin University. The authors also extend their gratitude SPEE3D for their generous support.]

We note that you have provided funding information that is not currently declared in your Funding Statement. However, funding information should not appear in the Acknowledgments section or other areas of your manuscript. We will only publish funding information present in the Funding Statement section of the online submission form.

Please remove any funding-related text from the manuscript and let us know how you would like to update your Funding Statement. Currently, your Funding Statement reads as follows:

[This research was supported by the Department of Education Australia through the Department of Education’s National Industry PhD Program (NIPhD 35017). The views expressed herein are those of the authors and are not necessarily those of the Australian Government or the Department of Education. There was no additional external funding received for this study.]

Additionally, because some of your funding information pertains to commercial funding, we ask you to provide an updated Competing Interests statement, declaring all sources of commercial funding.

In your Competing Interests statement, please confirm that your commercial funding does not alter your adherence to PLOS ONE Editorial policies and criteria by including the following statement: "This does not alter our adherence to PLOS ONE policies on sharing data and materials.” as detailed online in our guide for authors http://journals.plos.org/plosone/s/competing-interests.  If this statement is not true and your adherence to PLOS policies on sharing data and materials is altered, please explain how.

Please include the updated Competing Interests Statement and Funding Statement in your cover letter. We will change the online submission form on your behalf.

3. Thank you for stating the following in the Competing Interests section:

[I have read the journal's policy, and the authors of this manuscript have the following competing interests: Steven Camilleri, Tien Tran, Andrew Duguid, Mathew Harbidge and Riyan Rashid are employees of SPEE3D. This has been declared in the authorship details.].

We note that one or more of the authors are employed by a commercial company: SPEE3D.

1. Please provide an amended Funding Statement declaring this commercial affiliation, as well as a statement regarding the Role of Funders in your study. If the funding organization did not play a role in the study design, data collection and analysis, decision to publish, or preparation of the manuscript and only provided financial support in the form of authors' salaries and/or research materials, please review your statements relating to the author contributions, and ensure you have specifically and accurately indicated the role(s) that these authors had in your study. You can update author roles in the Author Contributions section of the online submission form.

Please also include the following statement within your amended Funding Statement.

“The funder provided support in the form of salaries for authors [insert relevant initials], but did not have any additional role in the study design, data collection and analysis, decision to publish, or preparation of the manuscript. The specific roles of these authors are articulated in the ‘author contributions’ section.”

If your commercial affiliation did play a role in your study, please state and explain this role within your updated Funding Statement.

2. Please also provide an updated Competing Interests Statement declaring this commercial affiliation along with any other relevant declarations relating to employment, consultancy, patents, products in development, or marketed products, etc.

Within your Competing Interests Statement, please confirm that this commercial affiliation does not alter your adherence to all PLOS ONE policies on sharing data and materials by including the following statement: "This does not alter our adherence to PLOS ONE policies on sharing data and materials.” (as detailed online in our guide for authors http://journals.plos.org/plosone/s/competing-interests) . If this adherence statement is not accurate and there are restrictions on sharing of data and/or materials, please state these. Please note that we cannot proceed with consideration of your article until this information has been declared.

Please include both an updated Funding Statement and Competing Interests Statement in your cover letter. We will change the online submission form on your behalf.

4. When completing the data availability statement of the submission form, we note that you have provided the source of datasets that is used for your study. However, the DOI that you provided shows “DOI not found”, which suggests that the DOI may have not been activated yet, and will be possibly available on acceptance. We strongly recommend all authors decide on a data sharing plan before acceptance, as the process can be lengthy and hold up publication timelines. Please note that, though access restrictions are acceptable now, your entire data will need to be made freely accessible if your manuscript is accepted for publication. This policy applies to all data except where public deposition would breach compliance with the protocol approved by your research ethics board. If you are unable to adhere to our open data policy, please kindly revise your statement to explain your reasoning and we will seek the editor's input on an exemption. Please be assured that, once you have provided your new statement, the assessment of your exemption will not hold up the peer review process.

5. Please amend the manuscript submission data (via Edit Submission) to include author Tien Tran.

6. Please amend your authorship list in your manuscript file to include author Thi Thuy Tien Tran.

7. Please ensure that you refer to Figure 3 in your text as, if accepted, production will need this reference to link the reader to the figure.

If the reviewer comments include a recommendation to cite specific previously published works, please review and evaluate these publications to determine whether they are relevant and should be cited. There is no requirement to cite these works unless the editor has indicated otherwise.

Additional Editor Comments:

The manuscript needs the revisions as suggested by the reviewers.

[Note: HTML markup is below. Please do not edit.]

Reviewers' comments:

Reviewer's Responses to Questions

**Comments to the Author**

1. Is the manuscript technically sound, and do the data support the conclusions?

The manuscript must describe a technically sound piece of scientific research with data that supports the conclusions. Experiments must have been conducted rigorously, with appropriate controls, replication, and sample sizes. The conclusions must be drawn appropriately based on the data presented.

Reviewer #1: Partly

Reviewer #2: Yes

**********

2. Has the statistical analysis been performed appropriately and rigorously?

Reviewer #1: N/A

Reviewer #2: N/A

**********

3. Have the authors made all data underlying the findings in their manuscript fully available?

The PLOS Data policy requires authors to make all data underlying the findings described in their manuscript fully available without restriction, with rare exception (please refer to the Data Availability Statement in the manuscript PDF file). The data should be provided as part of the manuscript or its supporting information, or deposited to a public repository. For example, in addition to summary statistics, the data points behind means, medians and variance measures should be available. If there are restrictions on publicly sharing data—e.g. participant privacy or use of data from a third party—those must be specified.

Reviewer #1: Yes

Reviewer #2: Yes

**********

4. Is the manuscript presented in an intelligible fashion and written in standard English?

PLOS ONE does not copyedit accepted manuscripts, so the language in submitted articles must be clear, correct, and unambiguous. Any typographical or grammatical errors should be corrected at revision, so please note any specific errors here.

Reviewer #1: Yes

Reviewer #2: Yes

**********

5. Review Comments to the Author

Please use the space provided to explain your answers to the questions above. You may also include additional comments for the author, including concerns about dual publication, research ethics, or publication ethics. (Please upload your review as an attachment if it exceeds 20,000 characters)

Reviewer #1: - The authors must explicitly clarify how this work advances beyond existing studies—particularly whether the contribution lies in quantitative correlation, LightSPEE3D-specific processing, or integrated mechanical–electrical optimization.

- The conclusions strongly rely on particle velocity values and their relation to critical velocity; however, there is no clear description of how velocity was measured or validated

- Porosity reduction and bonding improvement lack quantitative validation; no measurements of porosity, inter-splat bonding, or oxide thickness and distribution are provided.

- The manuscript does not address residual stress, which is a key factor in interpreting tensile and fracture behavior.

- Strength–ductility trade-off is inadequately explained: lacks dislocation density validation, no strengthening models applied, and ultimate tensile strength stabilization remains mechanistically unclear.

- The study focuses only on spray pressure, but other important factors like gas temperature, powder size, stand-off distance, and nozzle design are overlooked, limiting broader applicability.

- In introduction section these references may be helpful:

# Recent Progress in 5D Printing: Processes, Materials, Applications, and Future Trends

#Improvement in corrosion resistance of AISI 316L stainless steel weld cladding using GTA remelting technique

# Sensitization studies on low cycle fatigue and cryogenic impact behavior of AISI 304 SS weldments

Reviewer #2: 1. The manuscript addresses an industrially relevant topic, but the novelty is moderate because the influence of spray pressure on cold-sprayed copper has been previously reported, and the present study should more clearly define its unique mechanistic contribution beyond existing pressure–property studies.

2. The study focuses only on spray pressure, while other strongly coupled deposition parameters (gas temperature, standoff distance, feed rate, nozzle traverse speed) are held constant and insufficiently discussed, limiting the broader applicability of the conclusions.

3. The justification for selecting only four pressure levels (30, 40, 50, and 60 bar) is not adequately explained, and intermediate values could strengthen the identification of the proposed optimum processing window.

4. The manuscript repeatedly attributes improved ductility to continuous dynamic recrystallization (cDRX), but this central claim is inferred indirectly from XRD broadening and SEM rather than being directly validated using EBSD or TEM.

5. The use of Williamson–Hall analysis alone to estimate crystallite size and dislocation density is insufficient for a strong mechanistic claim, especially in heavily deformed copper where instrumental broadening and residual stress can significantly affect peak width.

6. The discussion of dynamic recrystallization is overstated, as no direct grain orientation maps or recrystallized fraction measurements are presented to distinguish recovery, polygonization, and true recrystallization.

7. The hardness and conductivity trends are interesting, but the manuscript does not adequately explain the apparent trade-off between reduced hardness and improved tensile ductility using established strengthening models.

8. The conductivity values are reported close to bulk copper (up to 98.4% IACS), but the manuscript does not sufficiently discuss possible measurement uncertainty, surface effects, or anisotropy in eddy current testing.

9. The claim of “bulk-like plasticity” is somewhat overstated, as the tensile strength remains significantly lower than wrought copper and should be discussed more cautiously.

10. The fracture analysis is qualitatively described, but the SEM fractography would benefit from quantitative dimple size analysis or fracture surface area comparison to support the ductile transition claim.

11. The manuscript lacks porosity quantification, despite porosity being repeatedly invoked as a major reason for conductivity and ductility changes.

12. The role of oxide rupture is central to the bonding mechanism, yet no chemical surface analysis (e.g., XPS or EDS mapping) is provided to directly support oxide disruption or reduction with pressure.

13. Residual stress, which is highly relevant in cold spray deposits and can strongly influence ductility and cracking, is not measured or discussed.

14. The particle velocity measurements are useful, but the manuscript does not sufficiently discuss velocity distribution spread, particle size dependence, or deposition efficiency, all of which are important for interpreting process–structure relationships.

15. The industrial significance is promising, but the manuscript would be stronger if the authors discussed how the identified 50–60 bar processing window translates into cost, energy consumption, and scalability in industrial CSAM.

**********

6. PLOS authors have the option to publish the peer review history of their article (what does this mean?). If published, this will include your full peer review and any attached files.

If you choose “no”, your identity will remain anonymous but your review may still be made public.

**Do you want your identity to be public for this peer review?**  For information about this choice, including consent withdrawal, please see our Privacy Policy.

Reviewer #1: No

Reviewer #2: No

**********

[NOTE: If reviewer comments were submitted as an attachment file, they will be attached to this email and accessible via the submission site. Please log into your account, locate the manuscript record, and check for the action link "View Attachments". If this link does not appear, there are no attachment files.]

To ensure your figures meet our technical requirements, please review our figure guidelines: https://journals.plos.org/plosone/s/figures

You may also use PLOS’s free figure tool, NAAS, to help you prepare publication quality figures: https://journals.plos.org/plosone/s/figures#loc-tools-for-figure-preparation.

NAAS will assess whether your figures meet our technical requirements by comparing each figure against our figure specifications.

# Author comment: Author response to Decision Letter 1

**DOI:** 10.1371/journal.pone.0353072.r002

22 May 2026

Response to Reviewers

Manuscript Title: Optimizing Cold Spray Additive Manufacturing: Pressure-Driven Enhancement of Mechanical Performance

Manuscript ID: [PONE-D-26-18004]

We sincerely thank the reviewers for their constructive and insightful comments. These have significantly improved the clarity, rigor, and scope of the manuscript. All comments have been carefully addressed, and the manuscript has been revised accordingly. A detailed response is provided below.

Reviewer #1: - The authors must explicitly clarify how this work advances beyond existing studies—particularly whether the contribution lies in quantitative correlation, LightSPEE3D-specific processing, or integrated mechanical–electrical optimization.

Response:

We thank the reviewer for this important comment. The novelty and contribution of the study have been clarified in the revised Introduction and Abstract. The manuscript now explicitly highlights that the present work establishes a process, structure and property relationship linking particle velocity, XRD-derived microstructural evolution, electrical conductivity, and tensile behaviour within a LightSPEE3D CSAM system. In addition, the study identifies a transition regime between 40–50 bar associated with significant changes in bonding behaviour and multifunctional performance.

- The conclusions strongly rely on particle velocity values and their relation to critical velocity; however, there is no clear description of how velocity was measured or validated

Response:

The velocity measurement was described in section 2.3 “Velocity of powder particles out of nozzle measurement”. It has detailed description of the Oseir HiWatch CS2 particle velocimetry system, including laser pulse interval, image acquisition conditions, particle tracking methodology, and averaging procedure.

- Porosity reduction and bonding improvement lack quantitative validation; no measurements of porosity, inter-splat bonding, or oxide thickness and distribution are provided.

Response:

We agree that direct porosity and oxide quantification would further strengthen the study. In the revised manuscript, statements regarding porosity reduction and densification have been moderated and are now presented as inferred trends based on conductivity behaviour and fracture morphology rather than direct measurements. In addition, a dedicated “Limitations and Future Work” section has been added to acknowledge the absence of quantitative porosity measurements, interfacial oxide characterization, and inter-splat bonding analysis. Future work involving image analysis, X-ray CT, and high-resolution microscopy has been proposed.

- The manuscript does not address residual stress, which is a key factor in interpreting tensile and fracture behavior.

Response:

We thank the reviewer for this valuable observation. A discussion regarding the potential influence of residual stress on tensile behaviour and fracture characteristics has been added to the revised Discussion section. The manuscript now explicitly acknowledges that residual stress was not measured and that future work involving XRD residual stress analysis or neutron diffraction would provide further insight into stress–microstructure interactions.

- Strength–ductility trade-off is inadequately explained: lacks dislocation density validation, no strengthening models applied, and ultimate tensile strength stabilization remains mechanistically unclear.

Response:

The Discussion section has been substantially revised to improve the mechanistic interpretation of the strength–ductility relationship. The revised manuscript now incorporates Hall–Petch strengthening concepts, hardening, and recovery/recrystallisation-assisted deformation mechanisms to explain the observed reduction in hardness and yield strength alongside improved ductility. We have also moderated several mechanistic claims to avoid over-interpretation beyond the available experimental evidence.

- The study focuses only on spray pressure, but other important factors like gas temperature, powder size, stand-off distance, and nozzle design are overlooked, limiting broader applicability.

Response:

We thank the reviewer for this valuable comment. The revised manuscript has been updated to clarify that gas temperature and stand-off distance were controlled during deposition and are now explicitly reported in the Experimental Procedure section (gas temperature: 750 °C; stand-off distance: 20 mm and federate of 60g/s).

The present study intentionally focused on spray pressure as the primary independent variable in order to establish a clearer process–structure–property relationship between pressure, particle velocity, microstructural evolution, and multifunctional performance in cold-sprayed copper. Powder size distribution was described in the manuscript.

We acknowledge that other parameters including nozzle geometry also influence particle acceleration and bonding behaviour. However, several of these parameters are associated with proprietary industrial system configurations within the LightSPEE3D platform and therefore could not be fully disclosed. To address this limitation, the manuscript now explicitly states that the identified processing window is specific to the operating conditions of the present system and should not be interpreted as universally transferable to all cold spray systems.

Additional discussion regarding the influence of coupled process parameters has also been incorporated into the Discussion and Limitations sections of the revised manuscript.

- In introduction section these references may be helpful:

# Recent Progress in 5D Printing: Processes, Materials, Applications, and Future Trends

#Improvement in corrosion resistance of AISI 316L stainless steel weld cladding using GTA remelting technique

# Sensitization studies on low cycle fatigue and cryogenic impact behavior of AISI 304 SS weldments

Response:

We have added those references into the Introduction section of the manuscript to strengthen the novelty of the manuscript.

Reviewer #2:

1. The manuscript addresses an industrially relevant topic, but the novelty is moderate because the influence of spray pressure on cold-sprayed copper has been previously reported, and the present study should more clearly define its unique mechanistic contribution beyond existing pressure–property studies.

Response:

We appreciate this comment and have revised the Introduction to better clarify the novelty of the work. While previous studies have investigated spray pressure effects independently, the present study focuses on establishing an integrated correlation between particle velocity, XRD-derived microstructural evolution, electrical conductivity, and tensile behaviour in a CS system. The identification of a transition regime between 40–50 bar has also been emphasized.

2. The study focuses only on spray pressure, while other strongly coupled deposition parameters (gas temperature, standoff distance, feed rate, nozzle traverse speed) are held constant and insufficiently discussed, limiting the broader applicability of the conclusions.

Response:

The revised manuscript now explicitly acknowledges that the present study isolates spray pressure while holding other process parameters constant in order to establish a clearer process–property relationship. Additional discussion regarding the influence of gas temperature, feed rate, stand-off distance, and nozzle geometry has been added.

3. The justification for selecting only four pressure levels (30, 40, 50, and 60 bar) is not adequately explained, and intermediate values could strengthen the identification of the proposed optimum processing window.

Response:

The manuscript has been revised to clarify that the selected pressure range (30–60 bar) was intentionally chosen to span conditions near, below, and above the expected critical velocity regime for copper deposition. This enabled identification of the transition from weak bonding behaviour to improved metallurgical bonding.

4. The manuscript repeatedly attributes improved ductility to continuous dynamic recrystallization (cDRX), but this central claim is inferred indirectly from XRD broadening and SEM rather than being directly validated using EBSD or TEM.

Response:

We agree and have carefully moderated the discussion throughout the manuscript. Statements previously implying direct confirmation of cDRX have been revised to indicate that the observed behaviour is “consistent with” or “suggestive of” recovery/recrystallisation-assisted refinement mechanisms. The revised manuscript now explicitly states that EBSD or TEM would be required for direct validation for future suggestion.

5. The use of Williamson–Hall analysis alone to estimate crystallite size and dislocation density is insufficient for a strong mechanistic claim, especially in heavily deformed copper where instrumental broadening and residual stress can significantly affect peak width.

Response:

We acknowledge this limitation. Additional discussion has been added clarifying that Williamson–Hall analysis provides an estimation of crystallite size and microstrain based on peak broadening and does not independently distinguish between recrystallisation, recovery, or residual stress contributions.

6. The discussion of dynamic recrystallization is overstated, as no direct grain orientation maps or recrystallized fraction measurements are presented to distinguish recovery, polygonization, and true recrystallization.

Response:

The Discussion and Conclusion sections have been revised to reduce overstatement regarding dynamic recrystallisation. Mechanistic interpretations are now presented more cautiously and framed as indirect evidence consistent with severe plastic deformation and recovery/recrystallisation-assisted refinement processes.

7. The hardness and conductivity trends are interesting, but the manuscript does not adequately explain the apparent trade-off between reduced hardness and improved tensile ductility using established strengthening models.

Response:

We have expanded the mechanistic discussion using Hall–Petch strengthening concepts, Taylor hardening, and recovery-assisted strain accommodation mechanisms to better explain the simultaneous reduction in hardness and improvement in ductility/conductivity.

8. The conductivity values are reported close to bulk copper (up to 98.4% IACS), but the manuscript does not sufficiently discuss possible measurement uncertainty, surface effects, or anisotropy in eddy current testing.

Response:

The Materials and Methods section has been expanded to include calibration procedure, testing frequency, repeated measurements, and testing methodology using the Fischer Sigmascope SMP350. Additional discussion has also been added to the possibility of uncertainty associated with surface condition and anisotropy.

9. The claim of “bulk-like plasticity” is somewhat overstated, as the tensile strength remains significantly lower than wrought copper and should be discussed more cautiously.

Response:

We agree and have revised the wording throughout the manuscript. The phrase “bulk-like plasticity” has been replaced with more cautious terminology such as “substantially improved ductility approaching bulk copper behaviour.”

10. The fracture analysis is qualitatively described, but the SEM fractography would benefit from quantitative dimple size analysis or fracture surface area comparison to support the ductile transition claim.

Response:

We acknowledge this limitation and have added a statement in the revised manuscript noting that quantitative dimple size analysis would further strengthen the interpretation of fracture behaviour and should be considered in future work.

11. The manuscript lacks porosity quantification, despite porosity being repeatedly invoked as a major reason for conductivity and ductility changes.

Response:

We agree and have moderated all direct claims regarding porosity reduction. The revised manuscript now presents densification and improved bonding as inferred trends supported by conductivity and fracture morphology observations rather than direct measurements. This limitation is explicitly acknowledged.

12. The role of oxide rupture is central to the bonding mechanism, yet no chemical surface analysis (e.g., XPS or EDS mapping) is provided to directly support oxide disruption or reduction with pressure.

Response:

The manuscript has been revised to reduce overstatement regarding oxide rupture. The discussion now refers more cautiously to “possible oxide fragmentation/disruption” inferred from conductivity improvement and XRD trends. The need for future EDS/XPS characterization has also been acknowledged.

13. Residual stress, which is highly relevant in cold spray deposits and can strongly influence ductility and cracking, is not measured or discussed.

Response:

A dedicated discussion regarding the possible influence of residual stress has now been added to the revised manuscript, together with acknowledgment of this limitation and recommendations for future investigation.

14. The particle velocity measurements are useful, but the manuscript does not sufficiently discuss velocity distribution spread, particle size dependence, or deposition efficiency, all of which are important for interpreting process–structure relationships.

Response:

The revised manuscript now clarifies that reported particle velocities represent mean values and that actual deposition involves a distribution influenced by particle size and gas dynamics. We acknowledge that deposition efficiency and velocity spread were not quantitatively analysed in the present work.

15. The industrial significance is promising, but the manuscript would be stronger if the authors discussed how the identified 50–60 bar processing window translates into cost, energy consumption, and scalability in industrial CSAM.

Response:

The revised Discussion section has been expanded to discuss the industrial implications of the identified 50–60 bar processing window, including the balance between improved deposit performance and increased gas pressure/energy requirements in practical CSAM applications.

Attachment Submitted filename: Response to Reviewers.docx

# Peer review: Decision Letter 1

**DOI:** 10.1371/journal.pone.0353072.r003

**Authors:** Gaurav Arora, Gaurav Arora

18 Jun 2026

Optimising Cold Spray Additive Manufacturing: Pressure-Driven Enhancement of Mechanical Performance in Copper Deposits

PONE-D-26-18004R1

Dear Dr. Tran,

We’re pleased to inform you that your manuscript has been judged scientifically suitable for publication and will be formally accepted for publication once it meets all outstanding technical requirements.

Within one week, you’ll receive an e-mail detailing the required amendments. When these have been addressed, you’ll receive a formal acceptance letter and your manuscript will be scheduled for publication.

An invoice will be generated when your article is formally accepted. Please note, if your institution has a publishing partnership with PLOS and your article meets the relevant criteria, all or part of your publication costs will be covered. Please make sure your user information is up-to-date by logging into Editorial Manager at Editorial Manager® and clicking the ‘Update My Information' link at the top of the page. For questions related to billing, please contact billing support.

If your institution or institutions have a press office, please notify them about your upcoming paper to help maximize its impact. If they’ll be preparing press materials, please inform our press team as soon as possible -- no later than 48 hours after receiving the formal acceptance. Your manuscript will remain under strict press embargo until 2 pm Eastern Time on the date of publication. For more information, please contact onepress@plos.org.

Kind regards,

Gaurav Arora, Ph.D

Academic Editor

PLOS One

Additional Editor Comments (optional):

An acceptance has been suggested for the article by the respected reviewers.

Reviewers' comments:

Reviewer's Responses to Questions

**Comments to the Author**

1. If the authors have adequately addressed your comments raised in a previous round of review and you feel that this manuscript is now acceptable for publication, you may indicate that here to bypass the “Comments to the Author” section, enter your conflict of interest statement in the “Confidential to Editor” section, and submit your "Accept" recommendation.

Reviewer #1: All comments have been addressed

Reviewer #2: All comments have been addressed

**********

2. Is the manuscript technically sound, and do the data support the conclusions?

The manuscript must describe a technically sound piece of scientific research with data that supports the conclusions. Experiments must have been conducted rigorously, with appropriate controls, replication, and sample sizes. The conclusions must be drawn appropriately based on the data presented.

Reviewer #1: Yes

Reviewer #2: Yes

**********

3. Has the statistical analysis been performed appropriately and rigorously?

Reviewer #1: Yes

Reviewer #2: N/A

**********

4. Have the authors made all data underlying the findings in their manuscript fully available?

The PLOS Data policy requires authors to make all data underlying the findings described in their manuscript fully available without restriction, with rare exception (please refer to the Data Availability Statement in the manuscript PDF file). The data should be provided as part of the manuscript or its supporting information, or deposited to a public repository. For example, in addition to summary statistics, the data points behind means, medians and variance measures should be available. If there are restrictions on publicly sharing data—e.g. participant privacy or use of data from a third party—those must be specified.

Reviewer #1: Yes

Reviewer #2: Yes

**********

5. Is the manuscript presented in an intelligible fashion and written in standard English?

PLOS ONE does not copyedit accepted manuscripts, so the language in submitted articles must be clear, correct, and unambiguous. Any typographical or grammatical errors should be corrected at revision, so please note any specific errors here.

Reviewer #1: Yes

Reviewer #2: Yes

**********

6. Review Comments to the Author

Please use the space provided to explain your answers to the questions above. You may also include additional comments for the author, including concerns about dual publication, research ethics, or publication ethics. (Please upload your review as an attachment if it exceeds 20,000 characters)

Reviewer #1: All reviewer comments have been satisfactorily addressed. The revisions have improved the manuscript's clarity, technical quality, and presentation. I recommend acceptance for publication.

Reviewer #2: (No Response)

**********

7. PLOS authors have the option to publish the peer review history of their article (what does this mean?). If published, this will include your full peer review and any attached files.

If you choose “no”, your identity will remain anonymous but your review may still be made public.

**Do you want your identity to be public for this peer review?**  For information about this choice, including consent withdrawal, please see our Privacy Policy.

Reviewer #1: No

Reviewer #2: No

**********

# Editor report: Acceptance letter

**DOI:** 10.1371/journal.pone.0353072.r004

**Authors:** Gaurav Arora, Gaurav Arora

PONE-D-26-18004R1

PLOS One

Dear Dr. Tran,

I'm pleased to inform you that your manuscript has been deemed suitable for publication in PLOS One. Congratulations! Your manuscript is now being handed over to our production team.

At this stage, our production department will prepare your paper for publication. This includes ensuring the following:

* All references, tables, and figures are properly cited

* All relevant supporting information is included in the manuscript submission,

* There are no issues that prevent the paper from being properly typeset

You will receive further instructions from the production team, including instructions on how to review your proof when it is ready. Please keep in mind that we are working through a large volume of accepted articles, so please give us a few days to review your paper and let you know the next and final steps.

Lastly, if your institution or institutions have a press office, please let them know about your upcoming paper now to help maximize its impact. If they'll be preparing press materials, please inform our press team within the next 48 hours. Your manuscript will remain under strict press embargo until 2 pm Eastern Time on the date of publication. For more information, please contact onepress@plos.org.

You will receive an invoice from PLOS for your publication fee after your manuscript has reached the completed accept phase. If you receive an email requesting payment before acceptance or for any other service, this may be a phishing scheme. Learn how to identify phishing emails and protect your accounts at https://explore.plos.org/phishing.

If we can help with anything else, please email us at customercare@plos.org.

Thank you for submitting your work to PLOS ONE and supporting open access.

Kind regards,

PLOS ONE Editorial Office Staff

on behalf of

Dr. Gaurav Arora

Academic Editor

PLOS One
